# Kikuchi-Fujimoto Disease: A Case of Febrile Cervical Lymphadenopathy With Hematological Abnormalities

**DOI:** 10.7759/cureus.47533

**Published:** 2023-10-23

**Authors:** Dong Eun Lee, Min Gyoung Pak, Sung-Hyun Kim, Christopher Chandler

**Affiliations:** 1 Department of Acute Medicine, University Hospitals Sussex NHS Foundation Trust, Worthing, GBR; 2 Department of Pathology, Dong-A University College of Medicine, Busan, KOR; 3 Department of Internal Medicine, Dong-A University College of Medicine, Busan, KOR

**Keywords:** histiocytic necrotizing lymphadenitis, cervical lymphadenopathy, kikuchi-fujimoto disease, kfd, pyrexia of unknown origin (puo), necrotizing histiocytic lymphadenitis, kikuchi disease

## Abstract

Kikuchi-Fujimoto disease (KFD), or histiocytic necrotizing lymphadenitis, is a benign but rare disorder associated with febrile cervical lymphadenopathy in young adults. Here, we discuss a case of a young female patient presenting with left tender cervical lymphadenopathy that progressed bilaterally with a fever of unknown origin. Laboratory parameters showed persistent leukopenia, especially neutropenia, which fluctuated with the degree of symptom severity. Two months were taken to confirm the diagnosis of KFD based on the histological interpretation of the lymph node biopsy. Supportive management with analgesics and paracetamol formed the main treatment. This case highlights the challenges and importance of diagnosing KFD to exclude other serious conditions such as lymphoma, tuberculosis, or lupus lymphadenitis that share similar clinical manifestations as KFD.

## Introduction

Kikuchi-Fujimoto disease (KFD), known as histiocytic necrotizing lymphadenitis, is a rare self-limiting disorder characterized by cervical lymphadenopathy. The disease generally affects young females under 30 years of age [1]. The main clinical manifestations include tender lymph nodes, fever, and malaise. The diagnostic challenges with KFD exist due to the low incidence rate worldwide and its overlapping clinical presentations with serious conditions such as lymphoma, tuberculosis, and other autoimmune diseases. Misdiagnosis is common and has led to unnecessary intervention and treatment [2]. The management of KFD is mainly supportive. Therefore, a prompt diagnosis could help avoid excessive investigations and treatments and provide reassurance to patients. The case of KFD uniquely portrays the diagnostic challenge presented by the fluctuating nature of lymphadenitis and marked hematological parameter changes in relation to disease activity. As such, the case could provide further insight into early recognition of KFD and an update to the diagnostic approach.

## Case presentation

A 26-year-old East Asian female presented to her general practitioner with a four-week history of left-sided, enlarged, painful cervical lymph nodes with fever and malaise. Initially, the symptoms were mild and flu-like with a few enlarged lymph nodes in the left neck. The following week, the symptoms progressed with an increased number of involved lymph nodes, nocturnal pyrexia with night sweats, and rigors. Prior to her presentation, the patient was generally fit and well without significant background medical history and no family history of note. She recently traveled to South Korea but had no sick contacts.

Examination demonstrated low-grade fever (38.5°C), tachycardia, and multiple palpable lymph nodes in the left cervical chain. The nodes were soft, fluctuant, and tender. Examination of the ear and oropharynx was unremarkable. The patient received an empirical course of oral cephalosporin to cover for presumed streptococcal pharyngitis, which showed no improvement. A broad infection screen was undertaken, but no cause was identified. The baseline chest X-ray was normal. Ultrasound examination of the lymph nodes showed multiple, less than 1 cm lymph nodes in the left cervical and supraclavicular region with maintained hilum and increased vascularity, suggesting reactive adenopathy. A computed tomography (CT) scan of the body did not suggest a lymphoproliferative cause. Lymph node biopsy was considered but not performed at this point as other results were reassuring. The symptoms spontaneously resolved after two weeks, and the patient remained symptom-free for a further two weeks with partial resolution of adenopathy.

**Figure 1:**
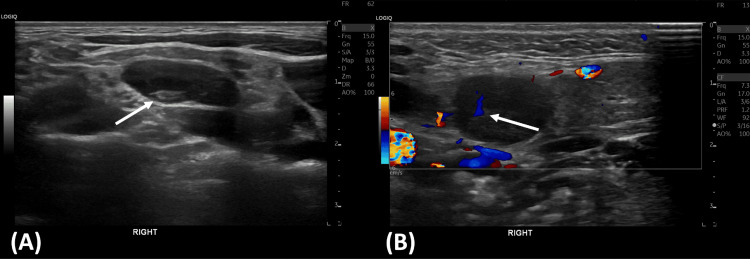
(A) Ultrasonographic imaging of the right cervical lymph node with maintained hilum (arrow). (B) Doppler ultrasonography showing a right cervical lymph node with maintained vascularity (arrow).

**Figure 2:**
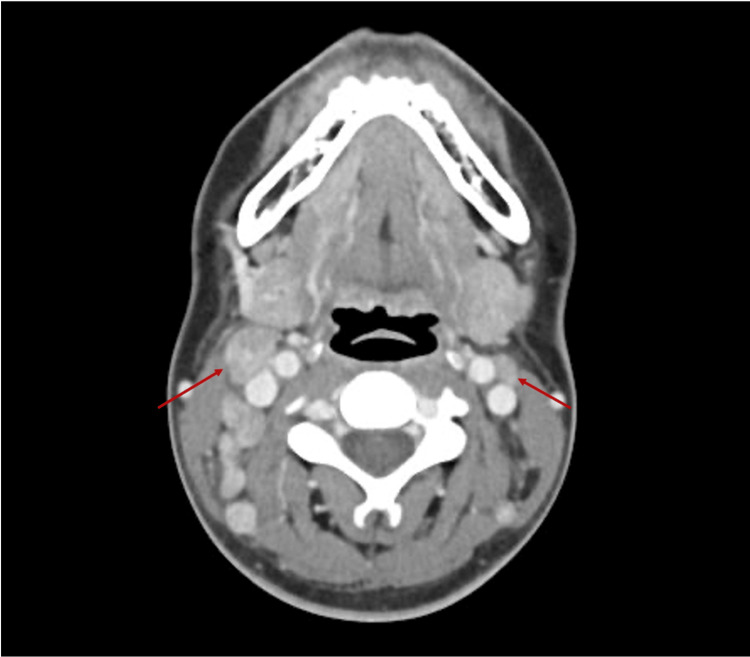
Second computed tomographic examination of the neck showing bilateral enhancing lymphadenopathy. Right cervical lymph nodes are more enlarged than left cervical lymph nodes (arrows).

Around week 8, the patient developed new painful lymph nodes in her right neck and both axillae. Fever and night sweats returned with new arthralgia in fingers. At this point, the patient had traveled to South Korea as a planned trip but was subsequently admitted due to worsening symptoms. On admission, she had moderate to severe neutropenia with fever and therefore received intravenous piperacillin and tazobactam in line with the febrile neutropenia protocol. Both ultrasound (US) and CT scans were repeated. US showed additional right cervical lymph nodes with the largest lymph node of 16×12 mm (Figure 1). CT imaging showed multiple enhancing lymph nodes bilaterally in cervical and supraclavicular regions and axillae with borderline splenomegaly (Figure 2).

**Figure 3:**
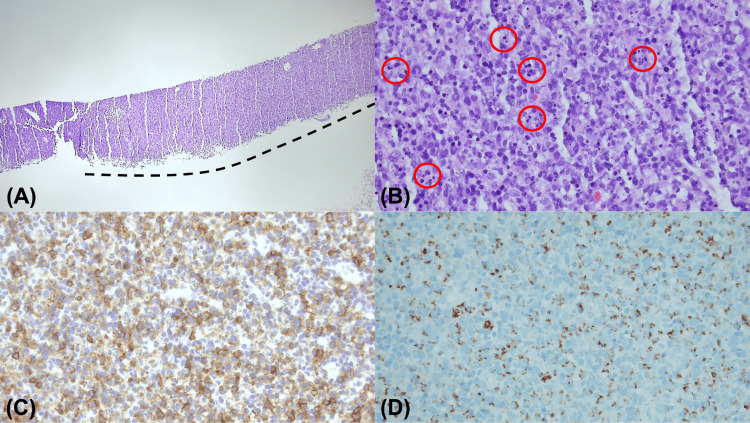
Pathology findings: (A) The core needle biopsy specimen from the right neck lymph node partially lost its normal histology and showed a poorly circumscribed pathological lesion (borders marked by dotted line) (hematoxylin and eosin stain, ×40 magnification). (B) Higher magnification of the lesion showed lymphohistiocytic infiltration with karyorrhectic debris (red circles) (hematoxylin and eosin stain, ×400 magnification). (C) The majority of the cells in the main lesion were positive for 5CD45RO (UTH-L1) immunohistochemical stain (CD45RO, ×400 magnification). (D) Crescentic histiocytes showed cytoplasmic positivity for CD68. (CD68, ×400 magnification).

Ultrasound-guided core needle biopsy (USCNB) of the right lymph node was performed, demonstrating histology consistent with a diagnosis of KFD. The affected lymph node was partially effaced by paracortical expansion. The main lesion was composed of lymphocytes, immunoblasts, and histiocytes, some with curved nuclei (crescentic histiocytes). There were abundant karyorrhectic nuclear debris and fibrin deposits. Neutrophils or eosinophils were not observed. Immunohistochemically, the infiltrates were mostly CD45RO (UTH-L1)-positive T-cells and CD68-positive histiocytes. CD20-positive B-cells were rare in the main lesion. The final diagnosis of histiocytic necrotizing lymphadenitis was made (Figure 3).

The overview of laboratory parameters is illustrated in Table 1. The initial results (week 4) showed mild leukopenia and neutropenia with slightly raised C-reactive protein (CRP). With aggravation of symptoms (week 5), white cell count (WCC) and neutrophil count became further deranged to 2.3×10^9^/L (normal range: 4-10×10^9^/L) and 0.8×10^9^/L (normal range: 2-7×10^9^/L), respectively. Inflammatory markers including CRP and ferritin were raised, along with lactate dehydrogenase (LDH). A peripheral blood smear showed a left shift of neutrophils with toxic granulation, red blood cells (RBCs) with rouleaux, and normal lymphocyte morphology. With the resolution of symptoms (week 6), cytopenia and LDH returned to baseline, although abnormal liver function tests (LFTs) were noted.

**Table 1:** Laboratory parameters over the duration of illness. ALT: alanine aminotransferase, AST: aspartate aminotransferase, ALP: alkaline phosphatase, eGFR: estimated glomerular filtration rate, CRP: C-reactive protein, LDH: lactate dehydrogenase, n/a: not available

Parameters	Week 4	Week 5	Week 6	Week 8 (admission)	Week 9 (follow-up)	Week 10	Normal range
Hemoglobin (g/L)	129	120	106	97	100	111	120-150
Mean corpuscular volume (fL)	90	89	90	86	87	87	84-101
Platelet (×10^9^/L)	196	190	267	143	180	199	150-410
White cell count (×10^9^/L)	3.1	2.3	3.1	1.5	1.4	3.2	4-10
Neutrophils (×10^9^/L)	1.5	0.8	1.3	0.5	0.6	1.5	2-7
Lymphocytes (×10^9^/L)	1.1	1.1	1.2	0.8	0.6	1.1	1-3
Monocytes (×10^9^/L)	0.5	0.3	0.4	0.2	0.2	0.4	0.2-1
ALT (u/L)	30	39	114	51	138	56	5-40
AST (u/L)	n/a	51	84	65	172	38	5-40
Albumin (g/L)	47	41	43	39	39	42	35-50
ALP (u/L)	73	77	72	74	82	74	20-150
Creatinine (µmol/L)	51	48	44	46	50	48	50-98
eGFR (mL/minute)	>90	>90	>90	>90	>90	>90	60-120
Sodium (mmol/L)	138	138	138	136	139	n/a	133-146
Potassium (mmol/L)	4.4	4.2	4	3.6	3.7	n/a	3.5-5.30
CRP (mg/L)	29	13	1	7	4	2	0-5
Ferritin (µg/L)	n/a	306	n/a	325	n/a	n/a	15-200
LDH (iu/L)	n/a	440	270	897	n/a	383	125-220

During the second manifestation of the symptoms (week 8), pancytopenia was evident, with prominent leukopenia and borderline severe neutropenia: WCC was 1.5×10^9^/L (normal range: 4-10×10^9^/L), neutrophil count was 0.5×10^9^/L (normal range: 2-7×10^9^/L), hemoglobin was 97 g/L (normal range: 120-150 g/L), and platelet count was 143×10^9^/L (normal range: 150-410×10^9^/L). LDH was further raised to 897 IU/L (normal range: 125-220 IU/L), and LFTs remained abnormal.

Comprehensive infection and autoimmune screens were performed, which showed unremarkable results. Blood cultures were negative. Viral serological screens for Epstein-Barr virus (EBV), cytomegalovirus (CMV), parvovirus, hepatitis B and C, and human immunodeficiency virus (HIV) infections were negative. Interferon-gamma release assay (IGRA) for tuberculosis was negative. *Bartonella henselae* serology was negative as well. Antinuclear antibody (ANA) titer, which was initially negative, became weakly positive with a dilution of 1:80. Other various tests including anti-double-stranded DNA (dsDNA) antibody, rheumatoid factor, antineutrophil cytoplasmic antibody, anti-Smith, anti-Ro/SSA, anti-La/SSB, and other markers for connective tissue disease were negative. Complement levels were normal, and the lupus anticoagulant screen test was negative.

The patient was admitted for five days with follow-up appointments. The treatment mainly consisted of a three-week course of regular paracetamol 650 mg three times daily and as required ibuprofen. Worsening transaminitis was noted during follow-up, but it quickly improved with the resolution of symptoms and discontinuation of paracetamol. In the outpatient follow-up, the symptoms were generally improved with residual lymphadenopathy, and the laboratory results returned to baseline. Lymph nodes were still enlarged at a six-month period but were non-tender and significantly reduced in size. There was no recurrence of KFD or evolution of other autoimmune conditions such as systemic lupus erythematosus (SLE) at the one-year follow-up.

## Discussion

KFD or histiocytic necrotizing lymphadenitis is a self-limiting disease that presents with tender lymphadenopathy and fever. It is an exceedingly rare condition with unknown incidence globally. It was first described in 1972 in two separate cases in Japan by Kikuchi and Fujimoto [2]. It is more prevalent in Asia; however, there is an increasing number of reports worldwide [2]. In a Korean study of 147 patients presenting with cervical lymphadenopathy, KFD was the most common diagnosis (34.7%), followed by tuberculous lymphadenitis (22.4%) [3]. In contrast, in an East Mediterranean study of 1,968 lymph node biopsies, only 0.6% were consistent with KFD [4]. Very few cases are available in Europe. In the United Kingdom, there were about 25 cases reported until 2018 [5]. KFD is thought to have a female predominance with a male-to-female ratio of 1:4, although recent literature describes equal sex distribution [6]. The age distribution ranges from six to 80 years; however, the mean age of presentation is in the 20s-30s [7].

The etiology remains poorly understood. Based on histopathological findings, the hyperimmune response of CD8+ lymphocytes and histiocytes to infectious agents has been proposed as the pathogenesis. EBV, CMV, human herpes virus (HHV)-6, HHV-8, parvovirus B19, hepatitis B virus, HIV, parainfluenza virus, *Yersinia enterocolitica*, and *Toxoplasma* have been listed as potential agents; however, their exact causative roles have not been established [8,9].

The majority of the patients present with unilateral cervical lymphadenopathy rather than bilateral nodal involvement. Most nodes are about 1-2 cm in diameter but could be as large as 7 cm [10]. Extra-cervical lymphadenopathy is not uncommon in regions such as the axillae, abdomen, inguinal, pelvis, or mediastinum. There is evidence that extra-cervical lymph node involvement is significantly associated with bilateral cervical involvement and leukopenia [11]. This is also evident in our case with the patient presenting with unilateral cervical lymphadenopathy initially, which progressed to bilateral cervical regions and axillae with worsening leukopenia. Fever is the next most common manifestation seen in about 30%-50% of patients, followed by rash, arthralgia, fatigue, and hepatosplenomegaly. Fever tends to persist for about a week, rarely up to a month [12]. Cervical lymphadenopathy has been shown to resolve without treatment within six months [13]. Recurrence as early as within 14 days has been reported and could illustrate the waxing and waning course of KFD as seen in this case [14].

In a Taiwanese study (n=367), 77% displayed abnormal full blood counts (FBCs), most commonly anemia (22%), followed by lymphopenia (17%), neutropenia (11%), and thrombocytopenia (8%). Cytopenia tends to be more prevalent in children rather than adults. The abnormal blood cell counts typically lasted about six months, rarely longer. The study also describes hemophagocytic lymphohistiocytosis (HLH), which is a life-threatening hyperinflammatory condition that presents similarly or secondary to KFD. HLH is associated with severe cytopenia, which typically involves more than two blood cell lineages. Bone marrow biopsy of HLH characteristically exhibits histiocytic infiltration, higher cellularity, and hemophagocytosis. In contrast, bone marrow examination of KFD describes hypocellular marrow with suppressed compensatory hematopoiesis [15]. Interestingly, our case presented with pancytopenia and borderline severe neutropenia, which initially raised a question of HLH. However, it was deemed unlikely as the clinical manifestations were insufficient to meet the diagnostic criteria, and there was an absence of hemophagocytic histiocytes in the peripheral blood smear. The exact mechanism remains unknown for deranged hematological parameters of KFD, but a mild degree of myelosuppression is the likely underlying mechanism as described in the study. Other non-specific laboratory findings associated with KFD include raised ESR, liver transaminitis, and high LDH. Autoimmune tests involving ANA, rheumatoid factor, or anti-double-stranded DNA are often negative. However, there are previous reports where initially negative ANA becomes positive with a new presentation of SLE [12].

Ultrasound examination of lymph nodes could be useful in distinguishing between benign and neoplastic causes of adenopathy. CT imaging also helps establish the extent of lymphadenopathy and the presence of hepatosplenomegaly. However, lymph node biopsy is the definitive investigation. There are various methods for the biopsy procedure. Historically, excisional biopsy (EB) has been the gold standard investigation. However, EB is invasive and may require general anesthesia and postoperative care. USCNB is another effective biopsy method, whereby a specimen is harvested with a biopsy needle under ultrasound guidance. Comparatively, this is a minimally invasive procedure and does not carry the potential aesthetic implications of EB. Recent studies have demonstrated a diagnostic accuracy of 95.6% in USCNB, suggesting that it is sufficient to serve as the primary diagnostic tool for KFD rather than EB [16,17].

KFD consists of three histological subtypes: early proliferative, necrotizing, and late xanthomatous phases. The proliferative phase is characterized by the presence of immunoblasts with crescentic histiocytes and karyorrhectic debris. The necrotizing phase shows mottled necrotic areas without neutrophilic infiltrate. The xanthomatous phase is represented by foamy histiocytes and fewer immunoblasts [18]. Our case describes the intermediate stage between proliferative and necrotizing phases. The histological differentiation of KFD can be challenging, especially if there are overlapping symptoms and serologies that suggest lupus lymphadenitis and non-Hodgkin lymphoma. The presence of extensive necrotic areas, hematoxylin bodies (dense homogeneous basophilic particles), or neutrophils would suggest lupus lymphadenitis in combination with serological tests. Cases with abundant immunoblasts could be mistaken for non-Hodgkin lymphoma, particularly mature T-cell lymphoma. Hence, it is necessary to perform immunohistochemical stains and B-cell/T-cell receptor gene rearrangement analysis [19]. Nonetheless, histological features are often sufficient to confirm the diagnosis especially if the pathologists are familiar with KFD.

There is no established single treatment for KFD. In severe cases, glucocorticoids or intravenous immunoglobulin have demonstrated efficacy. Otherwise, KFD has an excellent prognosis with most cases resolving within six months with a low recurrence rate of 3% [2]. However, a recent Korean study (n=102) reported that 20.6% of adult patients showed early or late recurrence with a disease-free interval ranging from 14 to 1,210 days. Extra-nodal features such as rash and positive ANA have been associated with a higher recurrence rate [14]. The relationship between KFD and SLE is well-established. Some suggest that KFD could be an early manifestation of SLE as the histological features of lymph nodes in these two conditions are indistinguishable. Some patients subsequently develop SLE; therefore, monitoring is recommended [2].

## Conclusions

KFD is a rare but benign disease, consisting of cervical lymphadenopathy and fever in young adults. This case highlights the importance of including KFD in the differential diagnoses in patients with febrile cervical lymphadenopathy where malignant and infectious causes have been excluded. Although a self-limited disorder, confirmation of the diagnosis remains crucial to exclude other serious diseases with confidence, to provide reassurance to both clinicians and patients, and to arrange appropriate follow-ups.
